# Correlation of Clinical and Pathological TNM Staging With Histopathological Grading in Oral Squamous Cell Carcinoma

**DOI:** 10.7759/cureus.60912

**Published:** 2024-05-23

**Authors:** Husna Tehzeeb, Alka Hande, Swati Patil, Archana Sonone, Aayushi Pakhale, Ankita Chavhan

**Affiliations:** 1 Oral Pathology and Microbiology, Sharad Pawar Dental College and Hospital, Datta Meghe Institute of Higher Education and Research, Wardha, IND

**Keywords:** head neck cancer, histopathological grading, tnm staging, prognosis, oral squamous cell carcinoma

## Abstract

Background

Oral squamous cell carcinoma (OSCC) is the most common type of head-neck cancer. The staging and grading of OSCC play an important role in disease management. Accurate staging helps in patient counseling, treatment planning, and prognostication in head-neck SCC. However, discrepancies between pathological and clinical staging have been stated, which affect disease prognosis.

Method

A retrospective review of 60 surgically treated patients with OSCC was done. Tumor-nodal-metastasis staging, both clinically and pathologically, was equated and tabulated to determine upstaging, downstaging, and cases where no stage change occurred. Additionally, the clinical and pathological TNM (tumor, node, metastasis) staging were correlated with the evaluation of histopathological grading.

Results

This study comprised 60 surgically operated OSCC patients. The T and N stages showed significant differences when compared clinically and pathologically. There was no significant correlation between histopathological grading and the disparities in TNM staging.

Conclusion

Some discrepancies exist between TNM staging evaluated clinically and pathologically for OSCC, which may show its effect on treatment planning and the prognosis of affected individuals. The histopathological analysis is the gold standard for the categorization of staging and grading in OSCC for proper treatment planning.

## Introduction

The most prevalent kind of cancer within the head and face area is oral squamous cell carcinoma (OSCC), which represents over 90% of cases of oropharyngeal and oral cancer [1]. Despite advanced techniques in treatment (surgery, radiation, chemotherapy), the survival of OSCC has not improved much. The chance of surviving five years is less than 50% worldwide [2]. Staging and tumor grading are critical components of OSCC management because they impact risk assessment and serve as a basis for individualized care [3]. The TNM (tumor, node, metastasis) approach to cancer staging depends upon evaluating the extent of the primary tumor (T), the presence of regional lymph nodes (N), which are infiltrated by malignant cells, and distant metastases (M) [4]. Treatment planning, recurrence prediction, and overall survival assessment all rely on this classification [5]. The American Joint Committee on Cancer (AJCC) published the initial staging guides in 1977. Two significant modifications were made to OSCC in the most current edition (AJCC eighth edition) in 2017. These were the addition of “extracapsular spread (ECS)” in the nodal stage and the inclusion of the “depth of invasion of a tumor (DOI)” in the T stage [6]. Various authors proposed histopathological grading in the literature [7-13]. In our study, we used Broders' classification, which graded OSCC as poorly, moderately, and well-differentiated histopathologically based on the variances in the resemblance of the tumor cells to parent tissue and the amount of keratin formed [14]. There have been observations of differences in staging when evaluated clinically and pathologically in head, neck, and face SCC. The difference between these stagings and the overall effect of this difference on histopathological grading have not yet been the subject of a single organizational study. To explore the effect of TNM staging differences on histopathological grading and the staging discrepancy rate in OSCC patients, we carried out a retrospective single-institute cohort study.

## Materials and methods

The current study is a retrospective investigation conducted at the Department of Oral and Maxillofacial Pathology and Microbiology. We retrieved the records of 60 patients who underwent surgical resection with neck dissection between 2019 and 2022 from the archives of this institute's department. The study was started with prior approval from the Datta Meghe Institute of Higher Education and Research's Ethical Committee (DMIHER (DU)/IEC/2023/1193). Patients with distant or recurrent metastases, as well as those who had received chemotherapy or radiotherapy before surgery, were excluded from the research. The demographic and clinical data were compiled, and pathological and clinical TNM stages were evaluated based on three parameters, which are the size of the primary tumor, nodal metastasis, and distant metastasis. Histopathologically, T stages were categorized into six stages, which are TX, Tis, T1, T2, T3, and T4. These T stages are based on the size and depth of the invasion (Table 1) [6].

**Table 1:** Primary tumor size (T stages) in TNM classification according to the AJCC eighth edition. Ca in situ: carcinoma in situ; DOI: depth of invasion; TNM: tumor, node, metastasis; AJCC: American Joint Committee on Cancer

T stages	Criteria for staging
TX	The tumor is not assessable.
Tis	Ca in situ.
T1	Tumor ≤2 cm and DOI ≤ 5 mm.
T2	Tumor ≤ 2 cm, DOI > 5 mm and ≤ 10 mm, or tumor > 2 cm and ≤ 4 cm and DOI ≤ 10 mm.
T3	Tumor > 4 cm or any tumor with DOI > 10 mm.
T4	T4a: When the tumor enters surrounding structures such as jaw bones or the skin of the face.
	T4b: Tumor invasion in facial spaces, pterygoid plates, skull base, or the internal carotid artery.

N stages are divided into NX, N0, N1, N2, and N3 stages, which are based on the number of lymph nodes involved and the extracapsular spread in regional lymph nodes (Table 2) [6].

**Table 2:** Classification of regional lymph node metastasis (N stages) according to the AJCC eighth edition. AJCC: American Joint Committee on Cancer

N stages	Criteria for staging
NX	Lymph nodes (LNs) are not assessable.
N0	Absence of metastasis in LNs.
N1	Involvement of single ipsilateral LN of size less than or equal to 3 cm without extranodal extension.
N2	N2a: Single ipsilateral LN of size 3-6 cm without extranodal extension.
	N2b: Multiple ipsilateral LNs ≤ 6 cm without extranodal extension.
	N2c: Contralateral or bilateral LNs of size ≤ 6 cm without extranodal extension.
N3	N3a: Involvement of LNs greater than 6 cm in size without extranodal extension.
	N3b: Involvement of LN(s) with extranodal extension.

And lastly, the distant metastasis is graded as M0 and M1 (Table 3) [6].

**Table 3:** Staging of distant tumor metastasis (M stages) according to the AJCC eighth edition. AJCC: American Joint Committee on Cancer

M stages	Criteria for staging
M0	Absence of distant metastasis.
M1	Distant metastasis assessed.

After that, based on TNM classification, the staging was ruled out in four stages: I, II, III, and IV (Table 4) [6].

**Table 4:** Various stages based on three parameters in the AJCC eighth edition classification. AJCC: American Joint Committee on Cancer

AJCC staging	T stages	N stages	M stages
Stage 0	Tis	N0	M0
Stage I	T1	N0	M0
Stage II	T2	N0	M0
Stage III	T1, T2, T3	N0, N1	M0
Stage IV			
Stage IV A	T1, T2, T3, T4a	N0, N1, N2	M0
Stage IV B	Any T, T4b	Any N, N3	M0
Stage IV C	Any T	Any N	M1

Subsequently, the patients were categorized into three groups according to Broders' histopathological grading system, which classifies them as having well, moderately, and poorly differentiated SCC. Lastly, these stages are correlated and tabulated to identify the discrepancies between them. Patients who were upstaged, downstaged, and patients with no stage change after the clinical and pathological comparison were divided into three groups.

## Results

Statistical analysis

Statistical analysis involves the application of descriptive and inferential statistics, including the Chi-square test. The analysis was conducted using the IBM SPSS Statistics for Windows, Version 27 (Released 2020; IBM Corp., Armonk, New York) software, with a significance level set at p<0.05.

Results

A total of 60 cases of OSCC were included, with a mean age of 50.81 years and a standard deviation of 13.20 years (ranging from 25 to 81 years). When evaluating gender, the majority of cases were male, accounting for 55 cases (84.62%). When examining clinical staging, the majority of patients were in stage IV A-20 (30.77%), followed by stage II-19 (29.23%), stage III-12 (18.46%), stage IV B-9 (13.85%), and only five cases in stage I (7.69%).

In our study, when the graph was plotted against the clinical T (cT) and pathological T (pT) stages, it showed significant results, as the p-value was 0.004. We observed that out of 60 patients, four (57.14%) who were in the T1 stage clinically were upstaged to the T2 stage pathologically, and five (20.83%) patients were upstaged from cT2 to pT4, whereas nine (33.33%) patients who were in the T4 stage clinically were downstaged to the T2 stage pathologically, and 13 (20%) patients were downstaged from cT4 to pT3 (Table 5).

**Table 5:** Correlation between clinical and pathological T stages showing significant results. T is the primary tumor size, which is the first parameter for the evaluation of TNM staging cT: clinical T stage; pT: pathological T stage

Clinical	pT1	pT2	pT3	pT4	Total
cT1	2 (28.57%)	4 (57.14%)	1 (14.29%)	0 (0%)	7 (10.77%)
cT2	3 (12.50%)	14 (58.33%)	2 (8.33%)	5 (20.83%)	24 (36.92%)
cT3	0 (0%)ƒ	3 (42.86%)	2 (28.57%)	2 (28.57%)	7 (10.77%)
cT4	1 (3.70%)	9 (33.33%)	8 (29.63%)	9 (33.33%)	27 (41.54%)
Total	6 (9.23%)	30 (46.15%)	13 (20%)	16 (24.62%)	65 (100%)
χ^2^-value	8.42, p=0.004, significant				

The graph was plotted against the clinical N (cN) and pathological N (pN) stages, and it does not show significant results, as the p-value is 0.21 (Table 6).

**Table 6:** Correlation between the clinical and pathological N stages, not showing significant results. cN: clinical N stages; pN: pathological N stages

Clinical	pN0	pN1	pN2	pN3	Total
cN0	12 (70.59%)	1 (5.88%)	2 (11.76%)	2 (11.76%)	17 (26.15%)
cN1	15 (68.18%)	0 (0%)	3 (13.64%)	4 (18.18%)	22 (33.85%)
cN2	11 (44%)	4 (16%)	7 (28%)	3 (12%)	25 (38.46%)
cN3	0 (0%)	0 (0%)	1 (100%)	0 (0%)	1 (1.54%)
Total	38 (58.46%)	5 (7.69%)	13 (20%)	9 (13.85%)	65 (100%)
χ^2^-value	11.92, p=0.21, not significant				

A statistical correlation was found between the histopathological grading and the pathological T staging. The statistical analysis (P= 0.013) showed that as the T stage advances, the tumor goes toward the higher grades (Table 7).

**Table 7:** A significant correlation was found between the pathological T staging and the histopathological grading. pT: pathological T stages; PDSC: poorly differentiated squamous cell carcinoma; MDSCC: moderately differentiated squamous cell carcinoma; WDSCC: well-differentiated squamous cell carcinoma

Pathological	PDSCC	MDSCC	WDSCC	Total
pT1	0 (0%)	2 (33.33%)	4 (66.67%)	6 (9.23%)
pT2	0 (0%)	17 (56.67%)	13 (43.33%)	30 (46.15%)
pT3	1 (7.69%)	7 (53.85%)	5 (38.46%)	13 (20%)
pT4	0 (0%)	14 (87.50%)	2 (12.50%)	16 (24.62%)
Total	1 (1.54%)	40 (61.54%)	24 (36.92%)	65 (100%)
χ^2^-value	6.11, p=0.013, significant			

There was no notable statistical correlation found between the histopathological grading and the pathological N staging (p=0.36) (Table 8).

**Table 8:** Correlation between the pathological N stages and the histopathological grades of OSCC. pN: pathological N stages; PDSCC: poorly differentiated squamous cell carcinoma; MDSCC: moderately differentiated squamous cell carcinoma; WDSCC: well-differentiated squamous cell carcinoma; OSCC: oral squamous cell carcinoma

Pathological	PDSCC	MDSCC	WDSCC	Total
pN0	1 (2.63%)	19 (50%)	18 (47.37%)	38 (58.46%)
pN1	0 (0%)	3 (60%)	2 (40%)	5 (7.69%)
pN2	0 (0%)	10 (76.92%)	3 (23.08%)	13 (20%)
pN3	0 (0%)	8 (88.89%)	1 (11.11%)	9 (13.85%)
Total	1 (1.54%)	40 (61.54%)	24 (36.92%)	65 (100%)
χ^2^-value	6.56, p=0.36, not significant			

## Discussion

OSCC, which predominantly manifests in the head, neck, and face area, stands as the most commonly encountered malignant tumor affecting the oral cavity [15]. The five-year survival rate for OSCC stands at approximately 60%, with minimal improvement over the past two decades despite notable progress in cancer detection and treatment methods [16,17]. The formulation of staging systems is carried out to compare patients with similar stages, which aids in prognosis assessment and offers valuable insights into the planning of further treatment [18]. Accurate staging is a crucial factor in determining the primary treatment and prognosis of OSCC patients [19]. The AJCC/International Union Against Cancer (UICC) staging system helps clinicians worldwide to stage cancer before treatment (cTNM), after surgical removal (pTNM), and upon recurrence (rTNM) [5]. The TNM approach of cancer staging considers three parameters, which are the extent of the primary tumor (T), the involvement of locoregional lymph nodes (N), and the presence of distant metastases (M) [20]. Discrepancies between clinical and pathological staging have been reported in head and neck squamous cell carcinoma (HNSCC). Analyses of these discrepancies are necessary for proper treatment planning for the patient.

This study was intended to correlate clinical and pathological TNM staging and to assess disparity in the same with different histopathological grades of OSCC, which showed a significant correlation between clinical and pathological T stages. This study builds upon a previous investigation conducted by Gupta et al. in 2015, which evaluated the incongruity between pathological and clinical TNM staging in patients with OSCC and its influence on survival. The researchers reviewed records of treated patients retrospectively and concluded that there are differences in the pathological and clinical TNM staging of OCSCC, which may have an impact on patient survival and treatment decisions. Therefore, a more uniform and consistent disease staging system is needed for appropriate decision-making [15].

In 2016, Kreppel et al. conducted another study on the comparison of the clinical significance of clinical histopathological staging in OSCC. In that retrospective study, a total of 392 patients with treatment-naive OSCC were included. They found that, despite advancements and contemporary radiologic techniques, pTNM outperforms cTNM in terms of predictive quality. Up to 40% of cases showed discrepancies between the clinical and histopathologic staging. Discordance between the staging of “clinical T and N stages” was more common than under-staging [21].

In 2003, Dantas et al. assessed the association between TNM classification and histological scores of malignancies, as well as the relationship between these parameters and prognosis, in 16 cases of tongue SCC. The information regarding the prognosis of patients and TNM classification (in a five-year follow-up) was analyzed, and the researchers concluded that while there was no correlation between the prognosis and the histopathological grades of malignancies, there was a significant correlation between TNM classification and prognosis. It was discovered that one important prognostic factor for tongue SCC was the TNM classification [22].

Another study by Rabie et al. in 2021 showed an association of nodal status clinically and pathologically in OSCC on 91 consecutive cases using the AJCC (eighth edition) staging system, the clinical (cN) along with pathological (pN) TNM nodal staging contrasted. By this, they concluded there is an unfortunate connection between clinical and pathological nodal conditions when the eighth TNM staging is applied. Extracapsular spread at the time of diagnosis is common in patients with OSCC [23]. The recent research revealed a noteworthy relation between clinical and pathological T stages. The latest AJCC TNM classification considers tumor size and depth of tumor invasion for evaluating the T stage, both of which require histopathological examination. Consequently, histopathological assessment plays a crucial role in determining the prognosis, lymph node metastasis, and disease-specific survival of patients with OSCC.

## Conclusions

The TNM staging system plays an essential role in determining the appropriate treatment approach for OSCC. Nonetheless, a notable number of patients diagnosed with OSCC based on their final pathological stage do not receive accurate clinical staging. The present study revealed a moderate level of agreement between the clinical and pathological T stages among patients with OSCC. Our results indicate that there are differences between pathological and clinical TNM staging in OSCC. Histopathological analysis is a more comprehensive and dependable method for classifying staging and grading in OSCC, aiding in accurate treatment planning. Pathological TNM staging independently influences the prognosis of OSCC patients. To ensure sufficient accuracy in T and N staging, it is crucial to standardize physical, radiological, and pathological examinations. Additionally, involving experienced radiologists and pathologists specializing in head and neck cancers is essential in the diagnostic process.
